# P106: Towards a new methodology in hygienic handrub testing

**DOI:** 10.1186/2047-2994-2-S1-P106

**Published:** 2013-06-20

**Authors:** M Wilkinson, DR Macinga, C Bradley, J Arbogast, B Okeke, F Brill, A Fraise

**Affiliations:** 1 University Hospitals Birmingham NHS Foundation Trust, Birmingham, UK; 2 Northeast Ohio Medical University, Rootstown, OH, USA; 3 GOJO Industries, Inc., Akron, OH, USA; 4 Dr. Brill + Partner GmbH, Hamburg, Germany

## Introduction

Internationally-recognized standards for evaluating alcohol based hand rubs (ABHR) differ significantly in methodology and success criteria. Hand hygiene authorities including the WHO and U.S. CDC have recognized inherent weaknesses with the current methods and have highlighted the need for improved *in vivo* efficacy methods, which reflect the use conditions and experience of healthcare practitioners.

## Methods

Variables within the European Standard EN1500 (Hygienic Handrub) method and ASTM standard E2755-10 method, including methods of hand contamination and modes of recovery, were tested on their ability to provide a consistent, robust method for testing ABHR.

## Results

The EN1500 method of contamination did not allow for hands to be sufficiently dry to adequately test smaller volumes of ABHR, typical of healthcare practitioner use. Whilst the ASTM E2755 method did allow for the hands to be sufficiently dry, the ‘glove-juice’ sampling technique of recovery was more cumbersome and led to a higher limit of detection, and thus potentially poor discrimination between products (2 x 3ml of 60% v/v propan-2-ol gave a mean log_10_ RF of 3.65; standard deviation 0.472). A hybrid method consisting of the ASTM E2755 method of contamination with an EN1500 method of recovery resulted in baseline recoveries of *Escherichia coli* K12 that were relatively low (mean log_10_ precount of 5.57; standard deviation 0.615). A hybrid method comprising the contamination of fingerpads with small volumes of microbial broth, coupled with the EN1500 method of recovery, appeared to yield a robust method that allowed dry hands to be tested with ABHR, with a low limit of detection (2 x 3ml of 60% v/v propan-2-ol gave a mean log_10_ RF of 5.62; standard deviation 1.108).

## Conclusion

Whilst further work is needed, it appears that a method involving the contamination of fingerpads, followed by the EN1500 method of recovery, may be a suitable candidate for inclusion in a single, globally-recognized *in vivo* efficacy standard, which would be more predictive of ABHR performance under clinical use conditions.

## Disclosure of interest

None declared

